# Genetic and Anatomic Determinants of Enzootic Venezuelan Equine Encephalitis Virus Infection of *Culex* (*Melanoconion*) *taeniopus*

**DOI:** 10.1371/journal.pntd.0001606

**Published:** 2012-04-03

**Authors:** Joan L. Kenney, A. Paige Adams, Rodion Gorchakov, Grace Leal, Scott C. Weaver

**Affiliations:** Institute for Human Infections and Immunity, Center for Tropical Diseases, and Department of Pathology, University of Texas Medical Branch, Galveston, Texas, United States of America; Centers for Disease Control and Prevention, United States of America

## Abstract

Venezuelan equine encephalitis (VEE) is a re-emerging, mosquito-borne viral disease with the potential to cause fatal encephalitis in both humans and equids. Recently, detection of endemic VEE caused by enzootic strains has escalated in Mexico, Peru, Bolivia, Colombia and Ecuador, emphasizing the importance of understanding the enzootic transmission cycle of the etiologic agent, VEE virus (VEEV). The majority of work examining the viral determinants of vector infection has been performed in the epizootic mosquito vector, *Aedes* (*Ochlerotatus*) *taeniorhynchus*. Based on the fundamental differences between the epizootic and enzootic cycles, we hypothesized that the virus-vector interaction of the enzootic cycle is fundamentally different from that of the epizootic model. We therefore examined the determinants for VEEV IE infection in the enzootic vector, *Culex (Melanoconion) taeniopus*, and determined the number and susceptibility of midgut epithelial cells initially infected and their distribution compared to the epizootic virus-vector interaction. Using chimeric viruses, we demonstrated that the determinants of infection for the enzootic vector are different than those observed for the epizootic vector. Similarly, we showed that, unlike *A. taeniorhynchus* infection with subtype IC VEEV, *C. taeniopus* does not have a limited subpopulation of midgut cells susceptible to subtype IE VEEV. These findings support the hypothesis that the enzootic VEEV relationship with *C. taeniopus* differs from the epizootic virus-vector interaction in that the determinants appear to be found in both the nonstructural and structural regions, and initial midgut infection is not limited to a small population of susceptible cells.

## Author Summary

Venezuelan equine encephalitis virus (VEEV) is transmitted to humans and horses by mosquitoes in Mexico, Central and South America. These infections can lead to fatal encephalitis in humans as well as horses, donkeys and mules, and there are no licensed vaccines or treatments available for humans. VEEV circulates in two distinct transmission cycles (epizootic and enzootic), which are differentiated by the ecological niche that each virus inhabits. Epizootic strains, those that cause major outbreaks in humans and equids, have been studied extensively and have been used primarily to develop and test several vaccine candidates. In this study, we demonstrate some important differences in the roles of different viral genes between enzootic/endemic versus epizootic VEEV strains that affect mosquito infection as well as differences in the way that enzootic VEEV more efficiently infects the mosquito initially. Our findings have important implications for designing vaccines and for understanding the evolution of VEEV-mosquito interactions.

## Introduction

Venezuelan equine encephalitis virus (VEEV) has been recognized as an etiologic agent of neurologic disease in humans and equids for nearly 80 years. Closely related to eastern (EEEV) and western equine encephalitis viruses (WEEV), VEEV belongs to the family *Togaviridae*, genus *Alphavirus*. First recognized in the 1920s, Venezuelan equine encephalitis (VEE) outbreaks are typically episodic with several years elapsing between outbreaks. However, when outbreaks do occur, they can cause severe and sometimes fatal disease in hundreds-of-thousands of equids and humans. For instance, after an interval of 19 years with no documented cases between 1973 and 1992, clusters of cases emerged in Venezuela [1] and Chiapas, Mexico [2] prior to a major outbreak involving ca. 100,000 people in 1995 [3]. In general, disease manifestations of VEE range from flu-like illness to fatal encephalitis. It is estimated that central nervous system (CNS) involvement occurs in 4–14% of human cases, and children are at the greatest risk to develop encephalitis and to die from infection [4].

Of the four subtypes of VEEV, IC and IAB are considered epizootic as they are known to cause disease in horses, to use these hosts for amplification, and are also capable of utilizing a variety of epizootic mosquito vectors, such as *Aedes* (*Ochlerotatus*) *taeniorhynchus*, *A.* (*Och.*) *sollicitans*, *Psorophora confinnis*, *Culex (Deinocerites) pseudes*, *Mansonia indubitans*, and *M. titillans*, among others [5]–[10]. Many of these mosquitoes thrive near coastal brackish water, can fly long distances from larval sites, prefer to feed on humans or other large mammals, and can tolerate feeding in sunny areas, although they may rest in shaded sites. In contrast, enzootic VEEV subtypes IE and ID generally cause little or no viremia or disease in equids, but like the epizootic strains, can cause fatal disease in humans [11]–[14]. Mosquito vectors that maintain these enzootic viruses in nature include a variety of species within the *Spissipes* section of the subgenus *Culex* (*Melanoconion*), and subtype IE strains specifically utilize *C. (Mel.) taeniopus*. The enzootic cycle typically occurs in shaded, intact forests with stable pools of water that are available for larval development. Some larvae also require the presence of a specific aquatic plant (i.e., *Pistia* spp.) for respiration [15].

Recent identification of extensive endemic disease in Peru, Bolivia, Ecuador, Colombia and Mexico, caused by spillover of enzootic strains in subtypes ID and IE, indicates the importance of VEEV as a continuous public health threat in Central and South America [16], [17]. The recent documentation of widespread endemic disease is likely associated with increased surveillance as well as the clearing of sylvatic forest habitats to accommodate the expansion of agricultural land types in areas of Latin America where enzootic VEEV persists [18]–[20]. The resulting fragmentation of sylvatic habitats results in an increase in ecotones that can support the life cycle of enzootic VEEV mosquito vectors [21], which also increases the likelihood of an enzootic VEEV strain adapting to epizootic transmission [22]. Enzootic ID strains are known to be a source for the emergence of epizootic IC strains and this emergence has occurred on multiple occasions [1], [23]. While IE strains had not been associated with the emergence of epizootic strains before 1993, recent outbreaks of epizootic-like IE strains were found to infect epizootic mosquito vectors and cause disease in equids [2], [24].

Historically, IE VEEV strains have been found in isolated sylvatic transmission cycles between *C. taeniopus* mosquitoes and rodent hosts, such as cotton rats (*Sigmodon* spp.), spiny rats (*Proechimys* spp.) and other rodent species, including *Liomys salvini* and *Oligoryzomys fulvescens* [25]–[27]. Phylogenetic studies of IE strains show that they diverged from other subtype I VEEV viruses, including enzootic ID strains [28], indicating that IE strains have long been established and most likely isolated within their enzootic habitats for at least centuries. Examination of the low threshold for infection and specificity of IE strains for *C. taeniopus* vectors suggests that IE stains have co-adapted to be highly fit for replication in and transmission by this vector [29]–[31].

**Figure 1:**
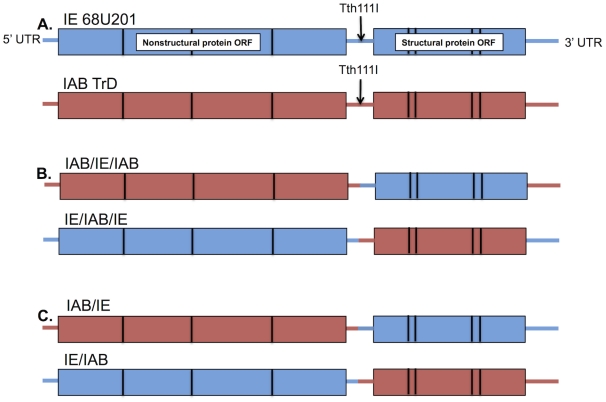
Schematic diagram of the virus strains utilized in this study. (A) The two parental viruses included strains IE 68U201 and IAB Trinidad Donkey (TrD). (B) IAB/IE/IAB and IE/IAB/IE were designed with matching *cis*-acting elements, which corresponded to the nonstructural protein opening reading frame (ORF) of the chimera. (C) IAB/IE and IE/IAB were designed with mismatched *cis*-acting elements, where the 3′ UTR matched the strain used for the structural protein region of the chimera and the 5′ UTR matched the strain used for the nonstructural protein region of the chimera. Tth111I indicates the location in which the two chimeric fusion fragments were joined by PCR.

The stable, enzootic VEEV IE-*C. taeniopus* relationship is in sharp contrast to the transient interaction that occurs between epizootic virus strains and mosquito vectors during sporadic outbreaks. However, the majority of experimental studies examining VEEV-vector interactions have utilized epizootic vectors as models. We hypothesize that IE viruses are highly adapted to their enzootic vector through a long-term evolutionary relationship such that the dynamics of infection of IE viruses within their vector differ inherently from those observed in epizootic virus-vector interactions. Reverse genetic studies of epizootic IC VEEV indicate that infection determinants reside within the E2 glycoprotein gene [21], [24], [28], [32]. We hypothesized that the transient nature of the epizootic virus limits its infection determinants to a localized region of the genome to allow for rapid adaptation to a competent vector, whereas the enzootic infection determinants are not limited to a single region in the structural portions of the genome due to the long adaptation of the genome to infection and replication within *C. taeniopus*. To test this hypothesis, we generated four chimeric VEEVs (Fig. 1), using a strain with a known high susceptibility to *C. taeniopus* (i.e., subtype IE strain 68U201) and a strain known to be poorly infectious for *C. taeniopus* [i.e., subtype IAB Trinidad donkey (TrD) strain]. These chimeras allowed us to discern the contributions of the structural and nonstructural protein regions as well as the 3′ untranslated region (UTR) in infection and dissemination in *C. taeniopus*.

We also examined the initial midgut infection dynamics of the enzootic mosquito model as compared to what has been previously observed in the epizootic model with IC VEEV and *A. taeniorhynchus*. There is only a small population of VEEV-susceptible midgut cells in *A. taeniorhynchus*, and thus the midgut infection is initiated by a very small number of infected cells and presumably virions [32]. Evolutionary theory would suggest that a bottleneck in the population of replicating viral genomes might deleteriously affect viral fitness through Muller's ratchet [33]–[35]. However, epizootic strains might regain fitness through recombination [32]. While this is a plausible strategy for an epizootic virus, which only interacts transiently with its mosquito vector during an outbreak, the enzootic virus must maintain a certain level of fitness to persist in nature over centuries or longer and repeated bottlenecks would likely be highly detrimental. We therefore hypothesized that most or all midgut epithelial cells in *C. taeniopus* are susceptible and, therefore, the population of enzootic VEEV that infect the midgut epithelium does not undergo a severe bottleneck during the infection of the midgut. To examine this hypothesis, we utilized viral-like particles (VLP) to establish the number, distribution, and susceptibility of midgut epithelial cells initially infected in the IE enzootic model.

## Materials and Methods

### Ethical statement

This study was performed in strict accordance with the recommendations in the *Guide for the Care and Use of Laboratory Animals* of the National Research Council. The protocol was approved by the Institutional Animal Care and Use Committee of the University of Texas Medical Branch (IACUC Protocol # 0209068, approved July 13, 2010).

### Cell culture and viruses

**Figure 2:**
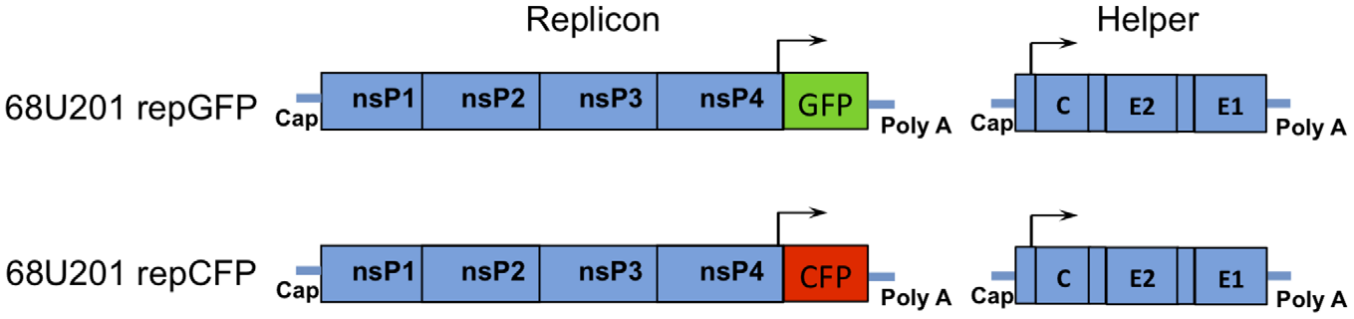
Schematic diagram of the replicons. (A) 68UGFP and (B) 68UCFP.

Plaque, cytopathic effect (CPE) assays, and replication curves were performed on Vero (African green monkey kidney), and BHK-21 (baby hamster kidney) cells were used for electroporation to rescue parental and recombinant viruses as well as replicon particles from transcribed RNA. Both cell types were propagated in Dulbecco's modified eagle medium (DMEM) supplemented with fetal bovine serum (FBS) and penicillin/streptomycin. For CPE assays of mosquito samples, amphotericin B (50 µg/mL) (Sigma-Aldrich, St. Louis, MO) was added to the DMEM. Cells from an *A. albopictus* mosquito cell line, C6/36, maintained in DMEM media supplemented with 10% FBS, penicillin/streptomycin, and 1% tryptose phosphate broth (Sigma-Aldrich, St. Louis, MO) were utilized for *in vitro* replicon co-infection experiments and replication curves. Viruses used for this study were derived from infectious cDNA clones V3000 IAB Trinidad Donkey (TrD) (kindly provided by Nancy Davis and Robert Johnston) [36] and IE 68U201 [37]. Prior to the generation of the V3000 clone, this TrD strain had been passaged once in guinea pig brains and 14 times in embryonated eggs. The 68U201 isolate had been passaged once in newborn mice and two times in BHK-21 cells prior to the construction of the clone. From these clones, four chimeric variants were developed: two with matching *cis*-acting elements and two with mismatched elements (Fig. 1B and C). Two IE replicons, 68UGFP and 68UCFP, were derived from a full length IE 68U201 clone as previously described (Fig. 2) [32]. Replicons are replication deficient VLPs that can be utilized to analyze the initial sites of infection without the complication of cell-to-cell spread. These particles were generated by electroporating two RNA species simultaneously. The replicon, consists of the nonstructural open reading frame expressing a fluorescent reporter and associated *cis*-acting elements; the helper contains the structural portions of the genome. Co-electroporation of these two RNAs generates deficient particles that are unable to package the structural genes, but continue to express only the nonstructural genes from the replicon packaged into the particle. Replicons and helpers were transcribed using a T7 mMessage mMachine (Ambion, Austin, Texas), electroporated into BHK-21 cells, and harvested after 24 hours.

### Generation of chimeric infectious clones

The first two chimeric clones were derived with mismatched *cis*-acting elements to directly compare the roles of the structural and nonstructural protein cassettes in mosquito infection and dissemination. Specifically, IAB/IE had the 5′ UTR and nonstructural protein gene region derived from IAB TrD and the structural protein gene region and 3′ UTR derived from IE 68U201. The reciprocal version, IE/IAB, had the 5′ UTR and nonstructural protein gene region of IE 68U201 and structural protein gene region and 3′ UTR derived from IAB TrD. Fusion PCR utilizing Phusion High Fidelity Polymerase (Finnzymes, Lafayette, CO) and designed around the Tth111I restriction enzyme site (Fig. 1) in the 26S UTR for each parental virus was used to generate a PCR fragment joining the two different viral cDNAs. Initially, for each reciprocal chimera, two overlapping fragments that encompassed the fusion site of the two genomes were generated by PCR using a forward primer from within the nsP4 region (7041 F IAB AND 6509 F IE) with a reverse fusion primer (IAB/IE R and IE/IAB R) and reverse primer downstream of the junction site (8007 R IAB and 8312 R) paired with a forward fusion primer for each chimera (IAB/IE F and IE/IAB R) (Table 1). The two individual fragments were joined by a PCR reaction on both templates utilizing the outermost primer sets. The fusion PCR fragment was cleaved with respective restriction enzymes (BssHII and PspOMI for IAB/IE and Bsu36I and NheI for IE/IAB) and ligated to the two other cDNA fragments with T4 DNA ligase (New England Biolabs, Beverly, MA). Ligated fragments were transformed into One Shot OmniMAX cells (Invitrogen, Carlsbad, CA), and resulting colonies were screened and sequenced prior to cesium chloride (CsCl) plasmid DNA purification.

**Table 1:** Primers used to generate chimeras.

Name	Description	Sequence
IAB/IE F	Tth111I Fusion Forward (A/E)	AACCTGAATGGACTACGACATAGTCAAGTCCGCCGAAATG
IAB/IE R	Tth111I Fusion Reverse (A/E)	CATTTCGGCGGACTTGACTATGTCGTAGTCCATTCAGGTT
7041 F IAB	Outer Joining Forward Primer (A/E)	AGCAGAGTGTTGAGAGAACGGC
8312 R IE	Outer Joining Reverse Primer (A/E)	TCATTCACTCCGCCAAGCAC
IE/IAB F	Tth111I Fusion Forward (E/A)	AACCTGAATGGACTGCGACGTAGTCTAGTCCGCCAAGATG
IE/IAB R	Tth111I Fusion Reverse (E/A)	CATCTTGGCGGACTAGACTACGTCGCAGTCCATTCAGGTT
6509 F IE	Outer Joining Forward primer (E/A)	GCTGCCCTGTATGCAAAGACTC
8007 R IAB	Outer Joining Reverse primer (E/A)	CTGAATAACTTCCCTCCGACCAC

Name	Description	Sequence
IAB/IE 3′UTR F	3′UTR Forward Fusion (A/E/A)	CCAACCAGAAACATAATTAGAATACAGCAGCAATTGGCAA
IAB/IE 3′ UTR R	3′UTR Reverse Fusion (A/E/A)	TTGCCAATTGCTGCTGTATTCTAATTATGTTTCTGGTTGG
10191 F IE	Outer Joining Forward (A/E/A)	AAATGCTGCGGCACTCAGG
12030 R	Outer Joining Reverse (Both)	AGGCACCCCAGGCTTTACACT
IE/IAB 3′UTR F	3′UTR Forward Fusion (E/A/E)	CCAACCAGAAACATAATTGATATTAGCAGCGATTGGCATG
IE/IAB 3′ UTR R	3′UTR Reverse Fusion (E/A/E)	CATGCCAATCGCTGCTAATATCAATTATGTTTCTGGTTGG
9528 F IAB	Outer Joining Forward (E/A/E)	CCGGAAAAGGGTGGGAGTTTG
12030 R	Outer Joining Reverse (Both)	AGGCACCCCAGGCTTTACACT

The IAB/IE and IE/IAB constructs were then utilized to generate the infectious clones with matching *cis*-acting elements: IAB/IE/IAB and IE/IAB/IE. For both IAB/IE/IAB and IE/IAB/IE chimeras, a fusion PCR was designed at the junction at the end of the structural protein gene region and the start of the 3′ UTR. As described above, two PCR amplicons were generated using primers 10191 F IE, IAB/IE 3′ UTR R, IAB/IE 3′ UTR F, and 12030 R for IAB/IE/IAB and 9528 F IAB, IE/IAB 3′ UTR R, IE/IAB 3′ UTR F, and 12030 R for IE/IAB/IE (Table 1). The two fragments were ligated and then cleaved with restriction enzymes (SpeI and SacII for IAB/IE/IAB and SgrAI and EcoRI for IE/IAB/IE) to generate a single cloning fragment. These clones were ligated in 3 fragments, transformed, purified, and sequenced as described above.

### *In vitro* transcription and RNA transfection

Prior to transcription, plasmids were linearized with either NotI (V3000 backbone) or MluI (68U201 backbone) restriction enzymes [37], [38]. RNA was generated using the mMessage T7 RNA Polymerase Kit in the presence of an analog cap (Ambion, Austin, TX). The yield and integrity of transcripts were evaluated by agarose gel electrophoresis directly prior to electroporation. BHK-21 cells were electroporated using previously described conditions [39]. Virus was harvested at 48 hours post-electroporation when CPE was observed in greater than 80% of the cells. Virus titers were determined by plaque assay on Vero cells.

### Viral replication analysis

Replication kinetics of each of the two parental strains and four chimera strains were compared on Vero and C6/36 mosquito cells to identify any deficiencies and compare to *in vivo* infection and dissemination in *C. taeniopus*. Cells were seeded at a concentration of 10^6^ cells/well in six well plates and allowed to attach and settle for 4 hours. Monolayers were infected in triplicate at a multiplicity of infection (MOI) of 5 PFU/cell and allowed to incubate for one hour at 37°C. Following incubation, cells were washed 3 times with phosphate-buffered saline (PBS), and overlaid with complete DMEM. Media were collected and stored from each well and replaced with the same volume of fresh media at predetermined time points, followed by plaque assays to measure viral yield. To compare the viral replication curves, a two-way ANOVA test and post-hoc multiple comparisons test with a Bonferroni correction was performed using JMP software, version 8.0.2 (SAS Institute Inc., Cary, NC). *P-values*≤0.05 were considered significant.

### Oral infection of mosquitoes

Two parental viruses (IAB, IE) and four chimeras (IAB/IE/IAB, IE/IAB/IE, IE/IAB, IAB/IE) were evaluated for their ability to infect and disseminate in *C. taeniopus*. The *C. taeniopus* colony was established from mosquitoes collected from Chiapas, Mexico in 2007 as described previously [40]. For all studies, 10-week old female CD1 mice (Charles River Laboratories) were used as viral hosts. To develop natural viremia, mice were infected with 3 log_10_ PFU of each virus [41] by subcutaneous (SC) inoculation, held for 24 hours, anesthetized by intraperitoneal (IP) administration of sodium pentobarbital (50 mg/kg), and bled via the retro-orbital sinus to determine viremia levels. Since the replicon particles utilized for this study do not replicate beyond the initial cell infected, and *C. taeniopus* will not feed on artificial bloodmeals, we utilized an artificial system in which we inoculated CD1 mice intravenously (IV) allowing for an immediate nonreplicative viremia. Mice were anesthetized by IP inoculation of sodium pentobarbital and 200 µl of a stock replicon or a 1∶1 mix of replicons was inoculated into the tail vein. Particles were allowed to circulate for 1–2 minutes before blood was collected from the retro-orbital sinus to estimate the artificial viremia level achieved; the animal was then exposed to mosquitoes for ca. one hour, after which blood was collected again from the retro-orbital sinus to detect any changes in the circulating replicon concentration. Following exposure, engorged mosquitoes were sorted and incubated for 14 days at 28°C with 75–80% humidity. A 10% sucrose solution was provided *ad libitum*. Statistical analysis of rates of infection and dissemination were broadly examined using a contingency analysis, and specific 2×2 comparisons were evaluated using Fisher's exact test with JMP software (SAS Institute Inc., Cary, NC). *P-values*≤0.05 were considered significant.

### Plaque and CPE assays

Viral titers of rescued viruses and animal sera were determined by plaque assay on Vero cells. Following the 14-day extrinsic incubation period (eip), legs and wings were removed from mosquitoes and stored at −80°C. Samples were triturated, centrifuged at 9500× G for 5 minutes, and used to infect monolayers of Vero cells in CPE assays. Triturated body samples that generated CPE were indicative of an infected mosquito, while legs and wings were used to detect a disseminated infection. Replicon titration was done in a similar manner to the plaque assay; ten-fold serial-dilutions were plated on a monolayer of Vero cells and allowed to incubate for one hour prior to an overlay with DMEM supplemented with FBS and penicillin/streptomycin. After 24 hours, the media were removed and the monolayer was fixed with 4% paraformaldehyde (PFA) (Affymetrix, Santa Clara, CA) for one hour. The number of fluorescent cells per well was counted using an Olympus Is71 inverted fluorescent microscope and reported as fluorescence units (FU).

### Midgut dissection and processing

Mosquito samples were processed 72 hours after blood feeding to minimize chances of damaging the midgut while distended with blood and to allow for clear images of the midgut epithelia. Mosquitoes were cold anesthetized and submerged for 30 seconds to 1 minute in 70% EtOH prior to being transferred to a PBS solution. Midguts were extracted and covered with a drop of 4% PFA on a glass slide for 30 minutes, then was rinsed twice with PBS before the addition of ProLong Gold Antifade with DAPI (Invitrogen).

### Microscopy

Mosquito midguts were imaged on an Olympus BX61 fluorescent microscope and high-resolution images were taken on an Olympus FluoView FV1000MPE confocal microscope. *In vitro* dual infection experiments were visualized on an Olympus DSU-IX81 spinning disk confocal microscope and analyzed with MetaMorph Software (Molecular Devices, Sunnyvale, CA).

## Results

### Viral replication kinetics

Figure 1 shows the genetic composition of the viruses utilized in this study. The two parental viruses included subtype IE strain 68U201 and subtype IAB strain TrD, which share 77% nucleotide and 89.8% amino acid identity. Four chimeric strains were derived from these parental strains: IAB/IE/IAB and IE/IAB/IE were designed with matching 5′ and 3′ *cis*-acting sequence elements. IAB/IE and IE/IAB were designed with mismatched *cis*-acting elements, where the 3′ UTR matched the strain used for the structural protein region of the chimera and the 5′ UTR matched the strain used for the nonstructural protein region of the chimera. It has been shown repeatedly that conserved regions in both the 5′ and 3′ UTRs of alphaviruses are essential for proper synthesis of both negative and positive strand RNA species [42]–[45]. Therefore, chimeras with both matching and mismatching *cis*-acting elements were utilized to compare these regions of interest and their effect on replicative efficiency.

**Figure 3:**
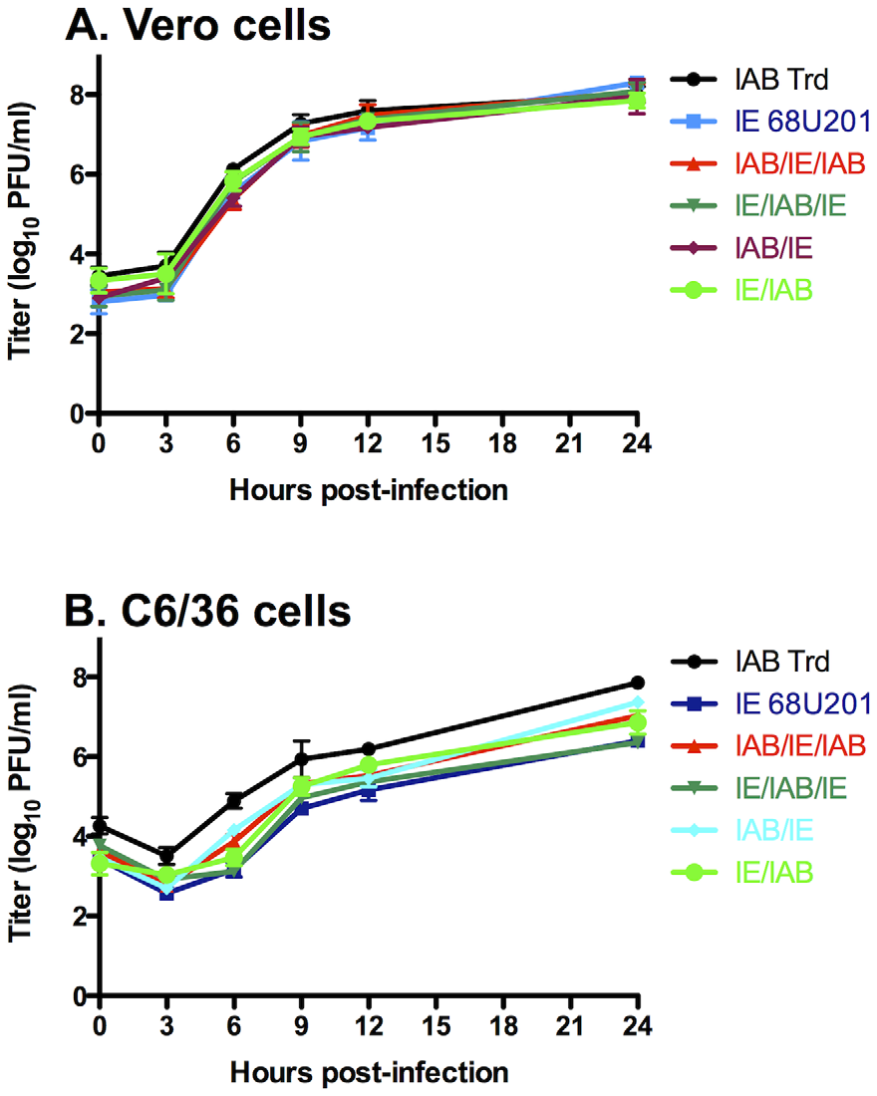
Viral replication kinetics. Comparison of two parental viruses IAB TrD and IE 68U201 and four chimeric viruses, IAB/IE/IAB, IE/IAB/IE, IAB/IE, and IE/IAB, at a multiplicity of infection (MOI) of 5 on Vero (A) and C6/36 (B) cells. Error bars represent the standard deviation of the means.

One-step replication curves of the parental and chimeric strains were performed on Vero cell monolayers at an MOI of five PFU/cell to identify any replication deficiencies that could bias experimental findings in the mosquito model. All of the chimeras showed similar replication, with no major deficiencies when compared to the parental strains (Fig. 3A). However, analysis of variance indicated that the replication of the viruses was significantly different (*p*<0.0001). Multiple comparison tests showed that strain TrD exhibited higher replication levels at multiple time points (not shown), which does not likely correlate to the *in vivo* mosquito model because this strain is unable to infect and disseminate in *C. taeniopus* [40], [46].

One-step replication analyses were also performed on monolayers of C6/36 *A. albopictus* cells to compare to the *in vivo* mosquito model (Fig. 3B). No major replication deficiencies were observed; however, analysis of variance did indicate differences among the viruses (*p*<0.0001). Unlike the Vero cell replication curves where just the TrD strain differed from all other viruses, differences were seen between nearly all viruses upon pairwise comparisons (not shown). The only three pairs out of the total 15 comparisons that did not show any statistical differences from one another were between IE versus IE/IAB/IE, IAB/IE/IAB versus IE/IAB, and IE/IAB/IE versus IE/IAB.

### Mosquito susceptibility to chimeric viruses

Adult female *C. taeniopus* were exposed to a range of oral doses for each of the parental and chimeric strains of VEEV and tested for infection and dissemination into the hemocoel following a 14-day eip (Table 2). Two pairs of chimeras with matched and mismatched *cis*-acting elements were utilized to independently evaluate the roles of the nonstructural and structural polyprotein open reading frames as well as the 3′ UTR in mosquito infection and dissemination. As expected, the parental IAB TrD virus was unable to infect *C. taeniopus* at blood meal titers as high as 6.2 log_10_ PFU/ml, which is in agreement with previous work [46], [47]. Similarly, as predicted based on previous studies [30], [40], [46], *C. taeniopus* mosquitoes were highly susceptible to infection with the parental subtype IE 68U201 strain at oral doses as low as 4.2 log_10_ PFU/ml.

**Table 2:** Virus infection and dissemination rates in *C. taeniopus*.

Virus	BM Titer	Infection rate [# infected/total # fed (%)]	Disseminated rate [# disseminated/total # fed (%)]	Dissemination infection rate [# disseminated/# infected (%)]
IAB	3.87	0/17 (0)	0/17 (0)	0/0 (0)
	5.11	0/18 (0)	0/18 (0)	0/0 (0)
	6.17	0/2 (0)	0/2 (0)	0/0 (0)
IE	4.18	13/16 (81)	7/16 (44)	7/13 (53)
	5.60	12/12 (100)	11/12 (92)	11/12 (92)
	5.72	3/3 (100)	3/3 (100)	3/3 (100)
IAB/IE/IAB	3.85	3/8 (38)	0/8 (0)	0/3 (0)
	4.81	0/16 (0)	0/16 (0)	0/0 (0)
	4.90	4/8 (50)	0/8 (0)	0/4 (0)
	5.77	2/4 (50)	0/4 (0)	0/2 (0)
	5.81	10/19 (53)	3/19 (16)	3/10 (30)
	8.50	13/23(57)	3/23(13)	3/13 (23)
	8.60	3/4 (75)	2/4(50)	2/3 (66)
IE/IAB/IE	3.20	0/10 (0)	0/10 (0)	0/0 (0)
	3.60	4/20 (20)	0/20 (0)	0/4 (0)
	3.98	9/17 (53)	0/17(0)	0/9 (0)
	5.30	1/4 (25)	0/4 (0)	0/1(0)
	6.00	5/16 (31)	2/16 (13)	2/5 (40)
	8.20	2/2 (100)	0/2 (0)	0/2 (0)
	8.40	16/20(80)	7/20(35)	7/16 (43)
IAB/IE	4.20	8/32 (25)	0/32 (0)	0/8 (0)
	4.70	7/20(35)	0/20(0)	0/7 (0)
	6.00	8/18 (44)	0/18 (0)	0/8 (0)
	6.23	2/22 (9)	0/22(0)	0/2 (0)
	8.30	10/20(50)	7/20(35)	7/10 (70)
	9.00	5/10 (50)	4/10 (40)	4/5 (80)
IE/IAB	4.60	8/27(30)	0/27(0)	0/8 (0)
	5.10	11/32 (34)	0/32 (0)	0/11 (0)
	5.50	22/24 (92)	7/24 (29)	7/22 (31)
	5.72	3/5 (60)	2/5 (40)	2/3 (66)
	8.20	30/34(88)	11/34(32)	11/30 (36)
	8.30	4/4 (100)	2/4 (50)	2/4 (50)

**Figure 4:**
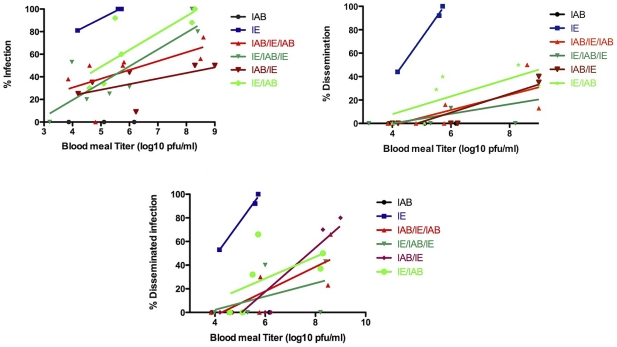
Oral exposure dose response of each virus examined during infection and dissemination in *C. taeniopus*. Regression lines were generated for each virus for the purpose of visualizing the results. Graph A represents the percentage of infection at a given dose. The goodness of fit R^2^ values were 0.995, 0.421, 0.725, 0.340, and 0.617 for IE, IAB/IE/IAB, IE/IAB/IE, IAB/IE, and IE/IAB, respectively. Graph B represents the percentage of dissemination at a given dose, which yielded R^2^ values of 0.996, 0.535, 0.465, 0.820, and 0.500 for IE, IAB/IE/IAB, IE/IAB/IE, IAB/IE, and IE/IAB, respectively. Graph C represents the percentage of disseminated infection, which resulted in R^2^ values of 0.992, 0.606, 0.2397, 0.838, and 0.305 for IE, IAB/IE/IAB, IE/IAB/IE, IAB/IE, and IE/IAB, respectively.

All four chimeras showed an intermediate ability to infect and disseminate in *C. taeniopus* when compared to the parental IAB and IE strains (Table 2; Fig. 4). The effect of the exposure dose on infection rate was evaluated by contingency analysis for each of the chimeric viruses (IAB/IE/IAB, IE/IAB/IE, IAB/IE, and IE/IAB) and found to be significant for each (*p*<0.05; *p*<0.001; *p*<0.05; *p*<0.0001, respectively) (Fig. 4A). In order to compare individual virus strains, a Fisher's exact test was utilized to determine differences in infection rates (Table 3). As expected, comparisons between the parental viruses and the chimeric viruses were all highly significant (*p*<0.0001), with the exception of the comparison between the IE parental virus and IE/IAB chimera, for which the IE strain had a less notable infectious advantage than the chimera (*p*<0.0071). Interestingly, infection rates did not differ significantly among three of the four chimeras: IAB/IE, IAB/IE/IAB, and IE/IAB/IE. However, IE/IAB showed a significantly higher infection rate when compared to each of the other three chimeras (*p*<0.0001; *p*<0.0037; *p*<0.0025, respectively).

**Table 3:** Two-tailed Fisher's exact tests for infection rates of *C. taeniopus*.

Virus	IAB	IE	IAB/IE/IAB	IE/IAB/IE	IAB/IE	IE/IAB
IAB	-					
IE	p<0.001	-				
IAB/IE/IAB	p<0.001	p<0.0001	-			
IE/IAB/IE	p<0.001	p<0.0001	NS^a^	-		
IAB/IE	p<0.001	p<0.0001	NS^a^	NS^a^	-	
IE/IAB	p<0.001	p<0.0071	p<0.0025	p<0.0037	p<0.0001	-

a NS, not significant.

Although each chimera showed the ability to disseminate into the hemocoel after midgut infection, the dissemination rates among the chimeras were low overall (Figs. 4B and 4C); therefore, no transmission experiments were performed. Previous studies of alphaviruses as well as other arboviruses have shown that infected mosquitoes often have virus restricted to the midgut, which is likely explained by a commonly recognized but poorly understood barrier to viral escape of the mosquito midgut [46], [48]–[50]. While dissemination rates increased as the exposure dose was increased, the overall rates of dissemination were too low to perform reliable statistical analysis. Fisher's exact tests comparing the rates of infected mosquitoes with dissemination (Fig. 4C), showed no differences between the four chimeras.

### *C. taeniopus* midgut infection

**Figure 5:**
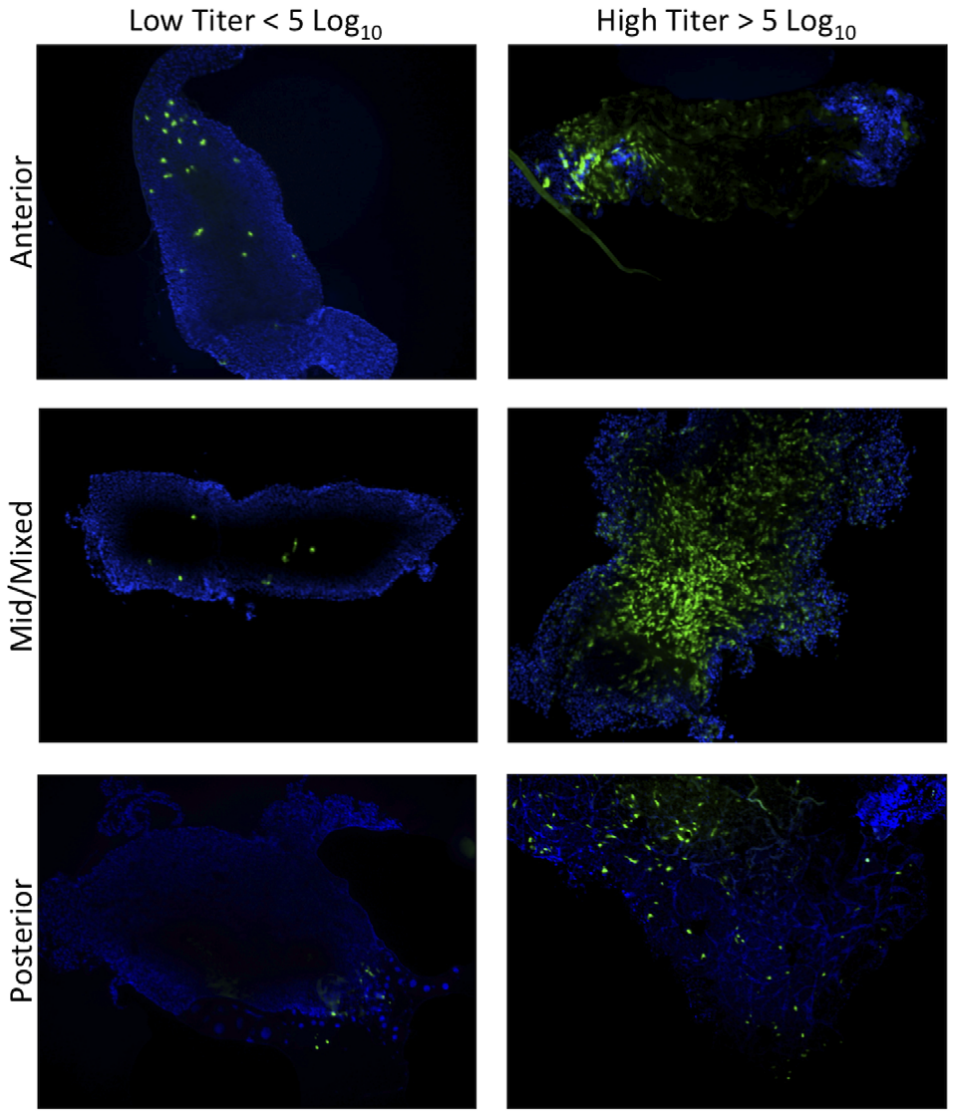
Sites of 68UGFP midgut infection in *C. taeniopus* (10×). Replicons were observed to infect the anterior, mid, and posterior portions of the abdominal midgut at all doses of exposure.

To observe the number of epithelial cells initially infected, the location of the infected cells, and to determine whether there is a subpopulation of cells within the midgut that is more susceptible than other epithelial cells, *C. taeniopus* mosquitoes were exposed to a range of doses of 68U201 replicon particles expressing fluorescent proteins (Fig. 2). For the single replicon infections, a clear dose-response was observed such that the lowest oral dose (average of pre- and post- exposure titers) of 3.0 log_10_ FU/ml infected only 11% of examined midguts with only 2 infected cells/midgut, whereas the highest dose of 7.2 log_10_ FU/ml infected 100% of examined midguts with a range 535–1757 infected cells/midgut (Table 4). Infected cells were not limited to any particular region of the abdominal midgut (Fig. 5), and only a minority of the midguts (9%) were found to have infection focused in the posterior portion, whereas 25% showed a focused infection in the anterior portion of the abdominal midgut. The remaining 66% of infected midguts showed a mixed infection with concentrated infection within the middle portion of abdominal midgut. Infection of the midgut/foregut junction was not observed.

**Table 4:** Initial midgut infection of 68U201 replicons in *C. taeniopus*.

Replicon Group	# midguts examined	Average experimental titer (FU)	# infected midguts(%)	# cells infected	Average # cells infected
68UGFP	9	3.00	1/9 (11)	2	n/a^a^
68UGFP	13	3.76	10/13 (77)	3–21	6
68UGFP	4	6.79	4/4 (100)	50–323	130
68UGFP	8	6.81	8/8 (100)	27–393	128
68UGFP	10	7.20	10/10 (100)	535–1757	1012

a n/a, not available.

### *C. taeniopus* midgut epithelial susceptibility

To determine if there was differential susceptibility of midgut cells, *C. taeniopus* mosquitoes were orally infected with a 1∶1 mixture of 68UGFP and 68UCFP. A total of fifteen mosquitoes was examined for co-infection at two different doses. The low exposure dose achieved by artificial viremia was a mixture of 5.4 log FU/ml 68UGFP and 5.0 log FU/ml of 68UCFP, and the high dose achieved was 6.5 log FU/ml of each replicon. At the low dose, an average of 70 midgut epithelial cells were infected with 68UGFP and an average of 52 cells was infected with 68UCFP. At the high dose, the average number of cells infected with 68UGFP was 896, whereas the average number of 68UCFP infected cells was 866. At the low dose and of the five co-exposed mosquitoes examined, no co-infected cells were observed. At the high dose where 15 midguts were examined, there appeared to be 2–3 cells with co-localization; however, it was determined that these areas of co-localization were a result of either signal bleed-through or overlap. Even so, there was still an average of less than one observed co-infected cell per midgut in the highest dose group.

## Discussion

As human populations continue to expand into rural environments, the incidence of emerging and re-emerging zoonotic pathogens will continue to climb. This has already been observed with other arboviral viruses, such as chikungunya, dengue, yellow fever, and Japanese encephalitis viruses [51]. Similar trends have also been observed with enzootic strains of VEEV that have caused endemic disease as well as outbreaks in Peru, Central America, and Mexico [16], [17]. Historically, studies of VEEV emergence have focused on epidemic strains within subtypes IAB and IC; however, enzootic ID and IE strains can also cause a large burden of endemic disease, which can often be misdiagnosed as dengue fever [52]. Recent studies have also shown that the primary mosquito vector of enzootic IE, *C. taeniopus*, can be an efficient vector of newly emerged epizootic IE strains in Mexico [40], [53]. Considering the growing risk of enzootic VEEV strains in causing human disease, it is important to understand the determinants and dynamics for viral infection of the primary enzootic vector, which we hypothesize to be different from what is known about the epizootic virus-vector interaction.

We first examined the genetic determinants of infection and dissemination utilizing chimeric viruses to analyze the molecular determinants for VEEV specificity to the enzootic mosquito vector, *C. taeniopus*. We used two viruses with distinct phenotypes for these chimeras, to help identify the major genome regions that contribute to specific infection of the enzootic vector. However, because these viruses have such a wide genetic divergence (10.2% at the amino acid level), extrapolation of this method to clarify the roles of each gene during enzootic mosquito infection could be prone to bias from incompatibilities between open reading frames within each chimera (Table 5). Therefore, to ensure that chimerization did not result in general attenuation of virus replication, the parental and chimeric strains were evaluated using *in vitro* replication curves on Vero and C6/36 cell monolayers; no replication deficiencies were observed. It was noted that the replication in mosquito cells was different from what was observed in the *in vivo* mosquito model in that the parental IAB virus, which showed no deficiencies *in vitro*, was unable to infect the *in vivo* model. Similarly, the differences observed between the IE parental and the four chimeras in the *in vivo* model were not demonstrated in the *in vitro* model. These results emphasize the importance of using an *in vivo* mosquito model to detect differences in viral replication, which may not be detected in a mosquito cell line.

**Table 5:** Identity percentage between the IAB TrD and 68U201 genomes.

5′ UTR (nt)	nsP1(AA)	nsP2(AA)	nsP3(AA)	nsP4(AA)	26S UTR (nt)
93%	95%	94%	69%	94%	86%
Capsid (AA)	E3 (AA)	E2 (AA)	6K (AA)	E1 (AA)	3′ UTR (nt)
86%	86%	87%	91%	93%	73%

We orally exposed *C. taeniopus* mosquitoes to varying doses of two parental strains, subtype IAB TrD and subtype IE 68U201, as well as four chimeric strains, IAB/IE/IAB, IE/IAB/IE, IAB/IE, and IE/IAB, and evaluated the role of the nonstructural and structural protein genes and the 3′ UTR as determinants of infection and dissemination in *C. taeniopus*. We hypothesized that, unlike the epizootic virus strains and their vectors, the genetic determinants for enzootic infection include multiple genes and they are not limited to a single region in the structural portion of the genome. Our data supported this hypothesis, as all four chimeras were able to infect and disseminate in *C. taeniopus*, albeit at rates lower than the wild-type IE parental strain. We included multiple replicates within each viral group to compensate for variations within the mosquito colony. If the E2 or the structural regions were the primary determinants of infection and dissemination, those chimeras with IE-derived structural regions would have infected mosquitoes at a higher rate than those chimeras with IAB-derived structural regions, and this was not observed.

We anticipated that the chimeras with mismatched 3′UTR regions would show diminished infection and dissemination rates based on previous alphavirus studies examining the effects of mismatched *cis*-acting elements [54], [55]; however, our IE/IAB chimera showed a significantly higher rate of infection than that of the other three chimeras. This suggests that the 3′ UTR plays some role in infection of the enzootic vector. A closer examination of the effect of the 3′ UTR on infection showed that the chimera with IAB structural genes and IAB 3′ UTR (IE/IAB) had the highest infection rate, while the chimeras with a mixed structural-3′ UTR makeup (IE/IAB/IE or IAB/IE/IAB) had intermediate infection abilities, and the chimera with IE in the structural and the 3′ UTR (IAB/IE) actually had the lowest rate of infection. This suggests that 3′ UTR acts in concert with other portions of the genome, although it is unclear which specific regions are important for this cooperative effect. These potential interactions should be further explored with 3′UTR-specific chimeras, such as a IAB virus backbone with a IE derived 3′UTR and a IE backbone with a IAB-derived 3′ UTR. While the role of these regions was not mirrored by *in vitro* mosquito infections, our C6/36 data were based on cells from *A. albopictus*, which in laboratory experiments has been shown to be equally susceptible to epizootic IC and enzootic ID VEEV strains [56]. Previous studies examining chimeras between Ross River virus (RRV) and Sindbis virus (SINV), two genetically distant alphaviruses, have shown that mismatched 3′ UTR regions can result in depressed RNA synthesis *in vitro*, although the effects on replication *in vivo* have not been examined [55]. However, studies of chimeras between more closely related alphaviruses, such as o'nyong-nyong (ONNV) and chikungunya (CHIKV) viruses, indicate that chimerization does not have a deleterious affect on the infection of the CHIKV mosquito vector, *A. aegypti*. There was also no indication that mismatched 3′ UTRs altered infection rates [57].

There were no statistical differences in the infection rates between chimeras IAB/IE, IAB/IE/IAB, and IE/IAB/IE, indicating that both the structural and nonstructural protein regions of the enzootic virus play a role in vector infection, as none of the chimeras displayed infection rates as high as the parental IE strain. However, we observed a trend in which the two chimeras with IE-derived nonstructural protein genes showed higher rates of infection at higher doses. Specifically, the chimeras with IE-derived nonstructural protein genes reached 100% infection at the highest doses, while the chimeras with IAB-derived nonstructural protein gene regions never reached 100% infection even at the highest doses. The diminished infection of all chimeras implies that there are multiple determinants of infection that reside in different genome regions and may act synergistically. Our results show that the determinants for infection of the enzootic vector do not reside solely in the structural protein genes, specifically not only in the E2 glycoprotein of the genome, which supports our hypothesis that infection determinants for VEEV in the enzootic mosquito vector relies on both structural or nonstructural protein regions of the genome. Interestingly, our findings suggest that in the enzootic model, the nonstructural elements are stronger determinants of vector infection.

We also hypothesized that the characteristics of initial midgut infection of the enzootic mosquito vector would be inherently different than those used by the epizootic virus in *A. taeniorhynchus*. To test this hypothesis, we exposed the enzootic vector, *C. taeniopus*, to replicon particles generated from a subtype IE enzootic strain. Examination of the initial sites of infection in the midgut indicated multiple locations in the abdominal portion with no predilection for either the anterior or the posterior region; we detected no infection of the cardial epithelium at the midgut/foregut junction. Similar to what was observed in *A. taeniorhynchus*, a clear response was observed between the oral dose and the number of infected midgut cells, although the ID_50_ for *C. taeniopus* was lower and the maximum number of infected cells was higher than the 100 susceptible *A. taeniorhynchus* cells previously estimated [32]. The greater number of infected cells (>1700) in *C. taeniopus* following high oral doses indicates that a larger number of its midgut epithelial cells is susceptible to VEEV IE infection compared to *A. taeniorhynchus* and VEEV IC. This observation, in conjunction with our observation of no co-infected midgut cells in the mixed replicon experiments, supports the hypothesis that the population size of enzootic VEEV virions during initial infection of the midgut is not severely restricted by a limited number of susceptible *C. taeniopus* epithelial cells. The average population of cells infected by the 68UGFP replicon at the highest dose did not differ from the average number of cells infected by the 68UCFP replicon. This suggests that there is no effect of co-exposure on individual particle infection rates. Utilizing the same methods, previous studies in the epizootic VEEV/mosquito model found an average of 26 midgut cells co-infected with two replicons, which was greater than what we observed in the enzootic model. This indicates that the initial infection of the enzootic vector differs from that of the epizootic VEEV strain. Using the Poisson distribution and given the epizootic data (a model with a small population of susceptible cells), we determined the probability of observing less than a single co-infected cell out of our five *C. taeniopus* midgut replicates to be 5.1×10^−12^, indicating an extremely low likelihood that there is a subpopulation of midgut epithelial cells with enhanced susceptibility.

Our studies illustrate the contrast in the virus-vector interactions between the enzootic and epizootic VEEV cycles. Not only do these interactions persist in different ecological cycles and infect different species of mosquitoes, but they also behave differently within their respective vectors. This difference may be explained by the dissimilar selective pressures that are exerted on each subtype during transmission. For example, epizootic viruses produce a very high level of viremia in equids, which facilitates VEEV transmission by epizootic vectors even if only a small population of their midgut cells are initially infected. However, the highly susceptible enzootic vector, which appears to have a greater number of susceptible midget cells that can be initially infected even after small oral doses, can transmit efficiently among populations of rodents that develop only moderate viremia titers [25]–[27].

As the growing impact of enzootic VEEV on human health is becoming more apparent, especially after the recent emergence of epizootic-like IE strains, understanding how these viruses interact with vectors is critical to estimating their threat to human health and for refining public health prevention strategies as well as developing vaccines. For instance, the design strategy of a vaccine that is protective against epizootic and enzootic strains that are currently causing human disease must also consider mosquito vectors that could potentially acquire and transmit should a vaccinee become viremic. If the epizootic vector only has a few susceptible midgut cells and is examined for competence for a given vaccine strain, it may appear to be incompetent. However, the same vaccine may be able to establish an infection in the enzootic vector. Considering that the determinants for infection appear to differ between the two vector types, vaccine strains that are derived from epizootic VEEV and depend on the elimination of mosquito infection may not necessarily reflect how infectious these vaccine candidates would be for enzootic vectors. As enzootic habitats are encroached upon and enzootic cycles gain close proximity to epizootic habitats, it is essential to consider the contribution of enzootic vectors to viral emergence and the potential introduction of vaccine strains into natural cycles.
